# Comparison of an Automated Plate Assessment System (APAS Independence) and Artificial Intelligence (AI) to Manual Plate Reading of Methicillin-Resistant and Methicillin-Susceptible Staphylococcus aureus CHROMagar Surveillance Cultures

**DOI:** 10.1128/JCM.00971-21

**Published:** 2021-10-19

**Authors:** Natalie Gammel, Tracy L. Ross, Shawna Lewis, Melissa Olson, Susan Henciak, Renee Harris, Ann Hanlon, Karen C. Carroll

**Affiliations:** a Division of Medical Microbiology, Department of Pathology, the Johns Hopkins Hospital, Baltimore, Maryland, USA; b The Johns Hopkins University School of Medicine, Baltimore, Maryland, USA; Mayo Clinic

**Keywords:** artificial intelligence, automated plate assessment system, MRSA, *Staphylococcus aureus*, CHROMagar

## Abstract

The automated plate assessment system (APAS Independence; Clever Culture System, Bäch, Switzerland) is an automated imaging station linked with interpretive software that detects target colonies of interest on chromogenic media and sorts samples as negative or presumptive positive. We evaluated the accuracy of the APAS to triage methicillin-resistant Staphylococcus aureus (MRSA) and S. aureus cultures using chromogenic medium compared to that by human interpretation. Patient samples collected from the nares on ESwabs were plated onto BD BBL CHROMagar MRSA II and BD BBL CHROMagar Staph aureus and allowed to incubate for 20 to 24 h at 37°C in a non-CO_2_ incubator. Mauve colonies are suggestive of S. aureus and were confirmed with latex agglutination. Following incubation, samples were first interrogated by APAS before being read by a trained technologist blinded to the APAS interpretation. The triaging by both APAS and the technologists was compared for accuracy. Any discordant results required further analysis by a third reader. Over a 5-month period, 5,913 CHROMagar MRSA cultures were evaluated. Of those, 236 were read as concordantly positive, 5,525 were read as concordantly negative, and 152 required discordant analysis. Positive and negative percent agreements (PPA and NPA, respectively) were 100% and 97.3%, respectively. The APAS detected 5 positive cultures that were missed by manual reading and determined to be true positives. In a separate analysis, 744 CHROMagar Staph aureus plates were read in parallel. Of these, 133 were concordantly positive, 585 were concordantly negative, and 26 required discordant analysis. PPA and NPA were 95.7% and 96.7%, respectively. This study confirmed the high sensitivity of digital image analysis by the APAS Independence such that negative cultures can be reliably reported without technologist intervention (negative predictive values [NPVs] of 100% for CHROMagar MRSA and 99.0% for CHROMagar Staph aureus). Triaging using the APAS Independence may provide great efficiency in a laboratory with high throughput of MRSA and S. aureus surveillance cultures.

## INTRODUCTION

Surveillance for indicator pathogens such as methicillin-susceptible Staphylococcus aureus and methicillin-resistant S. aureus (MRSA) is important for infection prevention in hospital settings (1, 2). One broadly used and relatively inexpensive surveillance method is to culture samples from the anterior nares of patients using chromogenic agars. As the pathogens of interest grow, an enzyme-substrate reaction occurs releasing chromogens in the agars (2–4). Various pigmented colonies, mauve, green, or another color depending upon the brand of medium used, are observed (2–4). As has been demonstrated in other studies, digital imaging software from automated platforms such as WASPLab can easily distinguish among pigmented colonies found on chromogenic agars and accurately separate positive from negative cultures (5–8).

The focus of this study, the automated plate assessment system (APAS) Independence (Clever Culture Systems, Bäch, Switzerland) is an automated system combining refined instrumentation that images, uses artificial intelligence (AI) to analyze the images, and subsequently categorizes samples in real time to determine the most appropriate designation as presumptive positive or negative (see Fig. S1 in the supplemental material). This system is capable of assessing a minimum of 200 plates per h with a colony size threshold of 0.5 mm. When linked with a laboratory information system (LIS), it has the potential to resolve negative samples independently. APAS is currently pending FDA clearance for MRSA cultures. The instrument relies on a software package known as an analysis module to interpret images and sort plates. The analysis module does not function independently of the APAS Independence instrument. Each analysis module is designed for the purpose of interpreting a specific solid medium for the presence or absence of a specific microbiologic target. We conducted a prospective study of the MRSA analysis module at the Johns Hopkins Hospital microbiology laboratory comparing manual reading of chromogenic MRSA and S. aureus surveillance cultures to the assessment by the APAS Independence.

## MATERIALS AND METHODS

### Study design and standard of care.

At the Johns Hopkins Hospital (JHH) and two of its affiliate institutions (Bayview Medical Center and Howard County General Hospital), all patients admitted to intensive care units (ICUs) and oncology units are screened weekly and on admission for MRSA colonization. In addition, the two neonatal intensive care units (NICUs) survey for both S. aureus and MRSA colonization. Anterior nares samples were collected using ESwab (Copan). All swabs were processed in the JHH microbiology laboratory. These swabs were inoculated onto BD BBL CHROMagar MRSA II and BD BBL CHROMagar Staph aureus (NICU patients only) using the Walk Away Specimen Processor (WASP). The WASP was programmed using a 10-μl loop, and the streak pattern was the 3-quadrant type 3 pattern. Plates were incubated according to the manufacturer’s recommendations for 20 to 24 h at 37°C in a non-CO_2_ incubator. After incubation, the technologist read the plates for the presence of a mauve-colored colony, and Staphylococcus aureus was confirmed by latex agglutination (Prolex Staph latex; Pro-Lab Diagnostics, Round Rock, TX). MRSA or S. aureus was reported as appropriate on the respective medium after confirmation.

### Operation of the APAS instrument.

**FIG 1:**
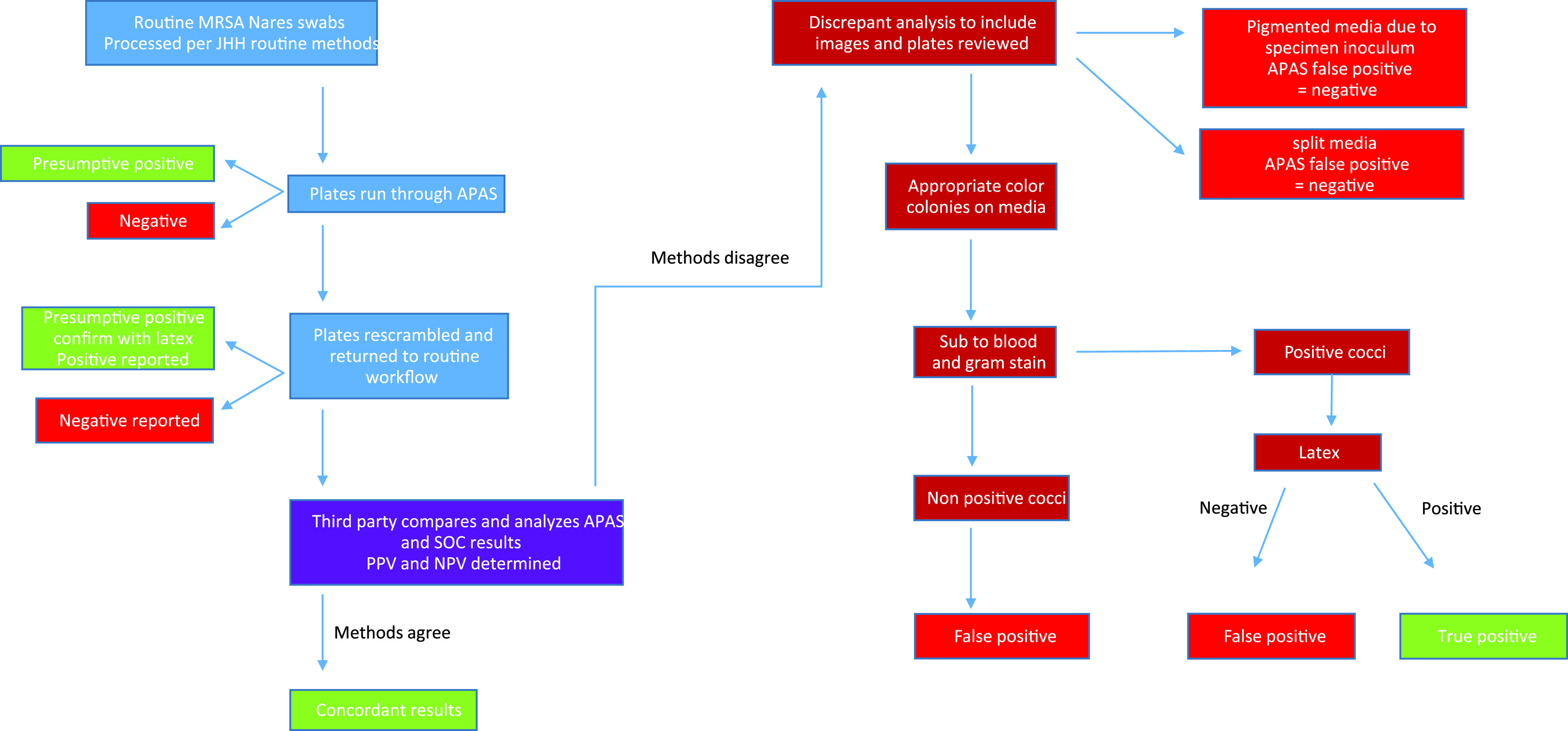
Workflow for the evaluation of the APAS interpretation compared to manual reading.

After incubation, the plates were loaded into the APAS instrument by a technologist that was not involved in the reading of the standard-of-care (SOC) method, and results were recorded by the instrument. This instrumentation includes a fully automated plate handling system that images the plates and then uses AI software (analysis module) to interpret the growth on the plate. The instrument will call a plate positive or negative based on the AI analysis and separates the plates into positive and negative stackers. After the APAS assessment of the plates, they were unloaded, rescrambled, and given to the technologist that was assigned to read the MRSA or S. aureus SOC manual method on that day (Fig. 1). The analysis module used in this study was the software designed specifically for reading BD BBL CHROMagar MRSA plates. Given the similarity of the BD BBL CHROMagar for both MRSA and S. aureus (identical agar compositions with the exception of cefoxitin), off-label investigations into the S. aureus performance were conducted.

### Data analysis and discordant result resolution.

Corresponding APAS and SOC results for each sample were compared and reviewed by a third-party reader. Initial positive percent agreement (PPA) and negative percent agreement (NPA) were determined for the APAS software digital assignment compared to the “truth” of the manual results. If the APAS assignments and SOC reading were in agreement, they were considered concordant. Any APAS and SOC sample set that was not in agreement was considered discordant. Both the CHROMagar plates and APAS-captured images were reviewed again, and the results were recorded. If a plate was flagged as discordant compared to the APAS digital assignment but found to only have a color-tinted inoculum on it, that plate was recorded as a negative result. Plates containing colonies of the appropriate color for the CHROMagar type had the colonies isolated to a blood agar plate for further analysis. Colonies of various hues similar to the appropriate color were also isolated. Plates were incubated for 20 to 24 h at 37°C in a non-CO_2_ incubator, and colonies were Gram stained. Isolates that were not Gram-positive cocci in clusters were recorded as negative for MRSA and/or S. aureus depending on the medium and considered to be false positives by the APAS system. Isolates that were Gram-positive cocci in clusters had a Staphylococcus latex agglutination test performed on them. Latex-positive isolates with a negative control were recorded as positive for S. aureus or MRSA as appropriate to the BBL CHROMagar plate from which it was isolated. Any isolate with a latex result that was questionable was further identified by matrix-assisted laser desorption ionization–time of flight mass spectrometry (Bruker MALDI Biotyper CA version 3.2, software Claim-4, 3.2.14; Bruker Daltonics, Billerica, MA). Isolates that were confirmed by rereading the CHROMagar plates following the procedure described above but missed on the initial read by the technologists were considered true positives.

## RESULTS

Over a 5-month period, 5,913 BBL CHROMagar MRSA plates cultured from 3,847 patients were read in parallel by the APAS Independence and by manual reading. Table 1 shows the performance of the APAS Independence compared to manual reading before discordant analysis. The data showed that 5,525 samples were concordantly negative and 388 samples were called presumptively positive by the APAS. Manual reading confirmed 236 of these to be true positives, while the other 152 required discordant analysis, for an overall agreement of 97.4%. The positive and negative percent agreements were 100% and 97.3%, respectively. The positive and negative predictive values were 60.8% and 100%, respectively.

**TABLE 1:** Comparison of the APAS Independence digital assignment to manual interpretation of BD BBL CHROMagar MRSA II and BD BBL CHROMagar Staph aureus

Medium	No. of specimens^*a*^					Value (% [95% CI])^*b*^			
	Tested	APAS+ MN+	APAS− MN−	APAS+ MN−	APAS− MN+	PPA	NPA	PPV	NPV
CHROMagar MRSA II	5,913	236	5,525	152	0	100 (96–100)	97.3 (97–97.5)	60.8 (59.3–62.3)	100 (96–100)
CHROMagar Staph aureus	744	133	585	20	6^*c*^	95.7 (92.7–98.7)	96.7 (94.5–98.9)	86.9 (80.7–93.1)	99 (92–100)

a APAS, APAS analytical module; MN, manual reading.

b PPA, positive percent agreement; NPA, negative percent agreement; PPV, positive predictive value; NPV, negative predictive value; CI, confidence interval.

c Upon rereview, one of these was misidentified by the technologist on manual read and is a true negative.

**FIG 2:**
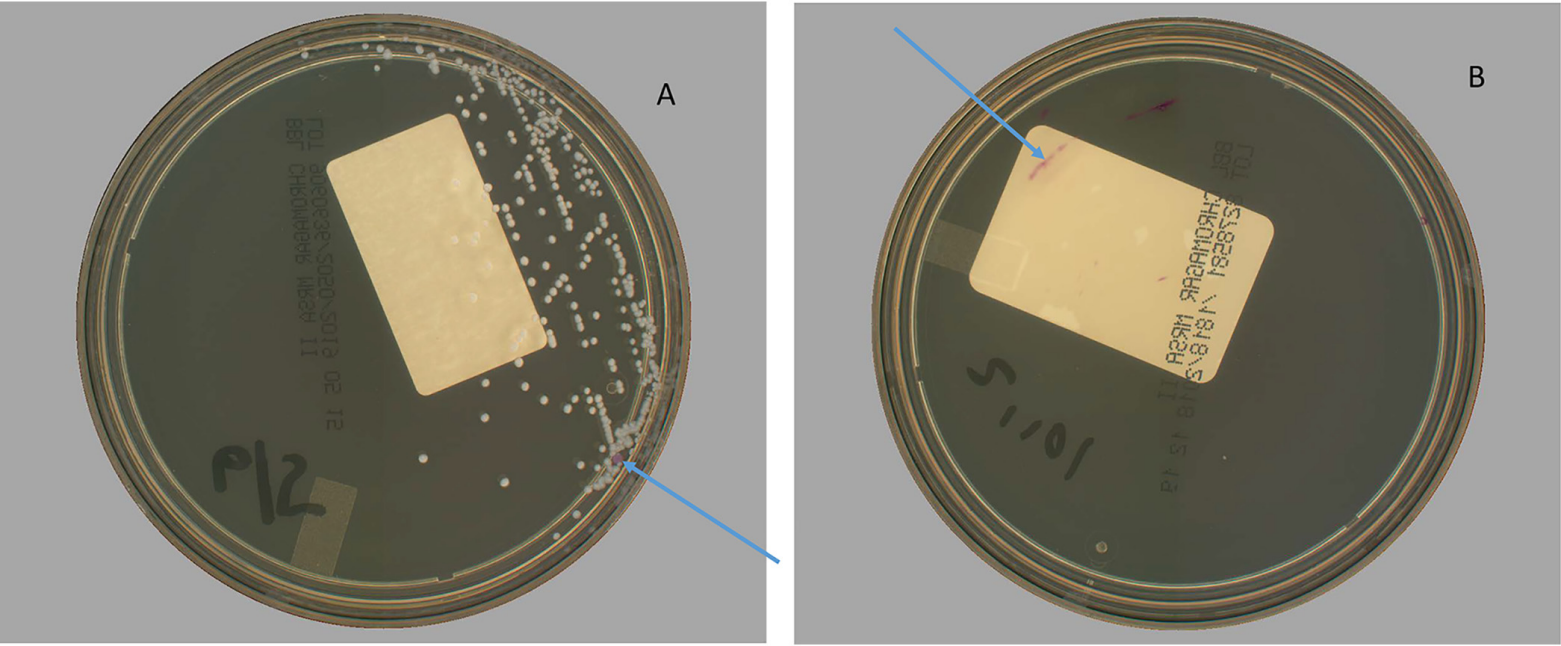
(A) An example plate with a single pink colony on the periphery that was missed by manual reading but detected by APAS Independence. (B) Pink-pigmented inoculum effect in the first quadrant sometimes seen with mucoid nares samples. The discolored/dim regions of the plates are known effects from the instrument’s camera, which do not affect any instrument function or algorithmic detection. (Photos are courtesy of Steven Giglio, LBT Innovations, Inc., Adelaide, Australia; reproduced with permission.).

Results of discordant analysis are depicted in Table 2. Interestingly, 3% of the discordant presumptive positives (*N* = 5) called by APAS were true MRSA missed by manual reading. In all cases, one or two pink colonies grew on the periphery of the medium in the initial area of inoculum and were missed by manual read (Fig. 2A). Of the remaining 147 cultures incorrectly flagged as positive, 85 (56%) demonstrated pink inocula (Fig. 2B) in the first quadrant, 44 (29%) had colonies similar in color to those of MRSA but were not S. aureus, and 18 (12%) were flagged due to defects in the CHROMagar, such as splitting or cracks (Table 2). The NPA improved to 97.4% after discordant analysis.

**TABLE 2:** Discordant resolution by category

Strain	No. of specimens with:^*a*^					
	Pink inoculum^*b*^	Positive manual read and negative APAS results	Pink colonies	False-positive manual read and negative APAS results	True positive missed by manual read	Agar issues
MRSA	85	0	44	0	5	18
S. aureus	10	5	5	1	5	0

a APAS, APAS analytical module.

b Pink inoculum was seen more frequently with traditional swab samples than with ESwab samples.

A total of 744 BD BBL CHROMagar Staph aureus plates were inoculated. Among these, 585 were concordantly negative, 133 were concordantly positive, and 26 were discordant, for an overall agreement of 96.5% (Table 1). The positive percent agreement and negative percent agreement were 95.7% and 96.7%, respectively, before discordant analysis. The positive and negative predictive values were 86.9% and 99.0%, respectively.

Results of the discordant analysis for the CHROMagar Staph aureus cultures are depicted in Table 2. Upon rereview of the 20 that were APAS positive but manual interpretation negative, five contained a small number of colonies that were missed by the technologist on the initial read. Among the remaining 15 false positives, 10 were due to pink inoculum in the first quadrant, and the remaining five plates had discrete pigmented colonies of various shades of pink. Regarding the six assigned by APAS as negative (APAS− MN+), one culture containing Gram-positive rods was entered incorrectly as S. aureus as part of the SOC. The remaining five cultures were false negatives by the APAS digital assignment. In these cases, the missed single colonies occurred in the initial area of inoculum that included more than usual normal flora and patient sample. After the discordant analysis, the PPA and NPA improved to 96.5% and 97.5%, respectively.

## DISCUSSION

At its core, microbiology laboratories rely on the visual skills of a technologist to manually read plates. This can be time consuming, inefficient, and subject to variation in results among readers, especially for complex polymicrobial samples (9). In one study by Glasson et al., the authors evaluated the agreement in interpretation of urine culture plate reading results among panels of microbiologists compared to consensus results defined as agreement between two or more panel member readings (9). Disagreement among the panelists with respect to enumeration of colonies on blood and MacConkey agars ranged between 1.5% and 5.5% (9). Others have noted that operator-dependent variables impacting the accuracy of disk diffusion susceptibility testing can be improved upon by automated plate streaking with inoculation devices such as the InoqulA BT (BD-Kiestra, Drachten, Netherlands) (10).

Automation is not new to clinical microbiology laboratories, and many microbiology laboratories have embraced the next level of instrumentation for specimen inoculation and “smart” incubation using total laboratory automation systems as a means of improving laboratory efficiency (11). Advanced automation now incorporates artificial intelligence into its functionality. Two of the automation manufacturers, Copan (WASPLab) and Clever Culture Systems (APAS Independence), have software that uses artificial intelligence, machine learning, and/or image analysis to provide an interpretation of colonies on chromogenic media (11). Several studies have highlighted the high negative predictive value of the WASPLab analysis software for interpretation of urine cultures and MRSA and vancomycin-resistant *Enterococcus* (VRE) surveillance cultures (5–8). In a high-throughput workplace, triaging samples in this manner consolidates the workload and reduces the need for technologist intervention on samples where critical thinking is not required, i.e., negative and no-growth plates.

There are several publications on the high accuracy of automated plate reading of urine cultures by the APAS system (12–14). In addition to our study, one other evaluation assessing a similar MRSA analysis module was reported by Aurbach et al. (15). In that large study of 17,000 specimens plated on chromID MRSA (bioMérieux, Marcy-l'Etoile, France), the sensitivity and specificity for known MRSA strains was 100%. There was a low number of false positives, as seen in our study, due to organisms other than S. aureus that demonstrated pigment (15). To our knowledge, this is the first publication evaluating the accuracy of the APAS MRSA analysis module on BD BBL CHROMagar and subsequent application to both MRSA and S. aureus CHROMagar. Compared to manual reading, the APAS demonstrated high accuracy and detected low-level positives missed by manual reading (2% to 3.6% of positives). Sensitivity ranged from 96% for CHROMagar Staph aureus to 100% for the CHROMagar MRSA, indicating a high negative predictive value such that “negative” cultures can be reliably reported without technologist review. It should be noted that the off-label application of the MRSA analysis module to the S. aureus screening samples demonstrated a lower specificity than for MRSA (by 4%). This is not an unexpected observation, as the development of AI algorithms rely upon suitable training data from a specific application for high-level performance. In the case of S. aureus screening, there is generally more breakthrough growth on the plate than that on MRSA screening plates, which would likely impact algorithm performance.

A critical piece to accurate reading by the APAS Independence’s analysis module is verifying the integrity of the solid medium. Agar should be free from cracks, splits, and large bubbles on the surface. Agar plates should also have no visible condensation on the medium or lid. Additionally, users must ensure adequate growth is achieved according to manufacturers’ specifications prior to loading onto the APAS Independence. Efficiency would be further enhanced if the Independence software was interfaced with the LIS for autoreporting of negative samples.

This study has a few limitations. This is a single center study and may not reflect the experience in other laboratories. In addition, the study design did not allow for an evaluation of the impact on technologist time compared to that for instrument reporting. Finally, we did not conduct a precision study in which a set of spiked specimens was read blindly by multiple techs and the APAS Independence. However, daily negative and positive quality control (QC) plate results passed consistently during the 85 days of the trial and were run by multiple operators.

In summary, this study demonstrated the ability of the APAS Independence to accurately discriminate positive from negative CHROMagar MRSA and Staph aureus cultures. The high negative predictive values indicate that cultures assigned as negative (78.6% for S. aureus to 93.4% for MRSA in this study) do not require additional technologist time. Because of the high false-positive screening rate, a technologist is still required to assess all plates flagged as positive. Additional studies of the APAS Independence assessing cost, reduction in turnaround time, and patient care benefits are needed.
