# Supplementary material for: Comparison of an Automated Plate Assessment System (APAS Independence) and Artificial Intelligence (AI) to Manual Plate Reading of Methicillin-Resistant and Methicillin-Susceptible Staphylococcus aureus CHROMagar Surveillance Cultures
Source: J Clin Microbiol. 2021 Oct 19;59(11):e00971-21. doi: 10.1128/JCM.00971-21 (PMC8525556; doi:10.1128/JCM.00971-21)
Supplement: Supplemental file 2 — Legend of Fig. S1. Download JCM.00971-21-s0002.pdf, PDF file, 0.03 MB [file jcm.00971-21-s0002.pdf]

Supplemental Figure 1: Photograph of the APAS Independence Instrument
